# A location-inventory model for the sustainable supply chain of perishable products based on pricing and replenishment decisions: A case study

**DOI:** 10.1371/journal.pone.0288915

**Published:** 2023-07-31

**Authors:** Zahra Mohammadi, Farnaz Barzinpour, Ebrahim Teimoury

**Affiliations:** School of Industrial Engineering, Iran University of Science & Technology, Tehran, Iran; Istinye University: Istinye Universitesi, TURKEY

## Abstract

In recent decades, changes in people’s lifestyles and occupations have led to new food consumption patterns around the world, with a notable growth in the demand for ready meals and meat products. Accordingly, the food industry has tried to transform the global food culture by promoting its more profitable products to become able to set a higher price for meat. Considering the short lifetime of products in perishable food supply chains, inventory decisions are considered crucial. In addition, the demand for perishable food products is greatly affected by their freshness. In this paper, we develop a multi-objective mixed-integer non-linear programming model for a four-level sustainable supply chain (SC) of a perishable product with price-dependent demand and deterioration rates. The SC consists of suppliers, a production center (PC), distribution centers (DCs), and retailers. We aim to ascertain the optimal pricing policy and cycle length to maximize profit, achieve specific social objectives, and minimize the total cost and adverse environmental impacts. The proposed model determines the deterioration rate of each product according to its expiration date, the optimal location of supply-side facilities, and the flow rate between the facilities based on the selected mode of delivery. The products are delivered to the retailers by a mechanized transportation system either directly from the PC or indirectly through the DCs. Finally, an actual case study is provided to demonstrate the applicability of the model and our theoretical results under real-world conditions. We solve the case study by a preemptive fuzzy goal programming method and perform several sensitivity analyses on the results. By performing simultaneous sensitivity analyses on the demand and expiration dates, the optimal values of the problem’s parameters are determined. The optimal values help decision-makers make optimal decisions regarding the selling price of products and replenishment times. The model is applicable to supply chains of any perishable items with expiration dates.

## 1. Introduction

**Fig 1:**
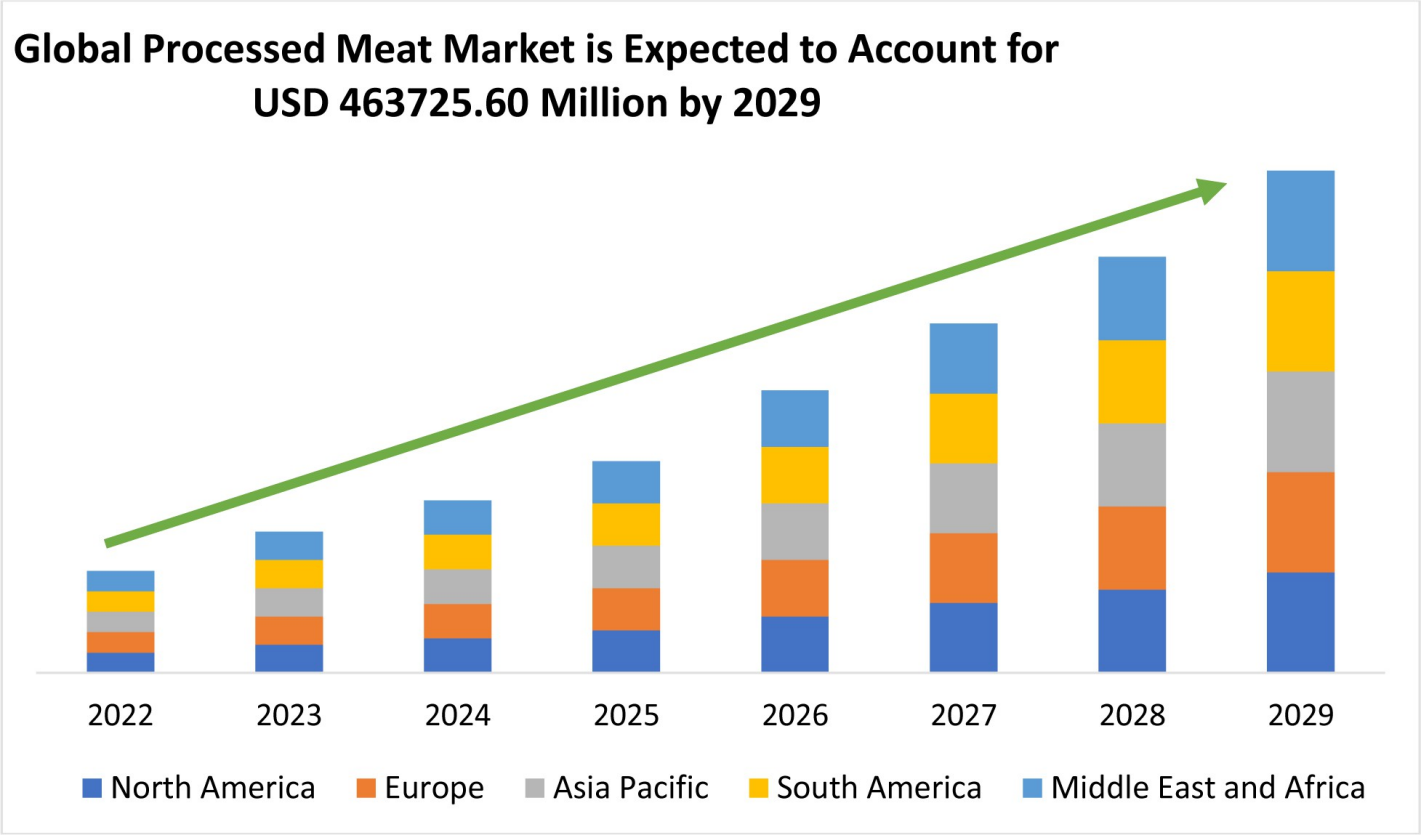
Global processed meat market by region (2022–2029) [2].

Today, the transformation of lifestyles and occupations have led to major changes in traditional patterns of food consumption around the world, with a greater tendency toward ready meals and processed meat products [1]. Fig 1 shows the current projections regarding the growth of the global demand for processed meat products [2].

**Fig 2:**
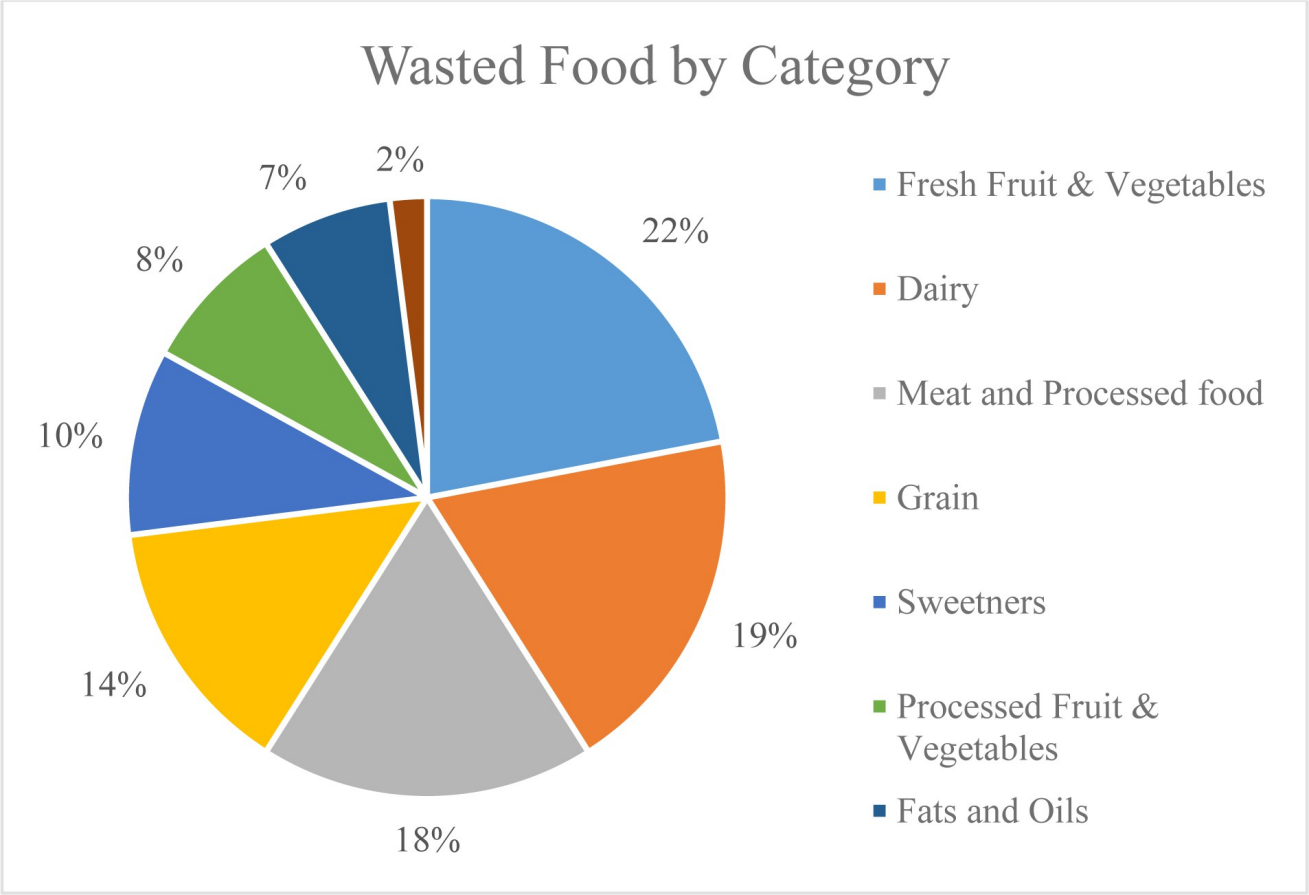
Largest sources of food waste in the world [2].

From a global perspective, food waste is one of the clearest manifestations of poverty and nations’ failure to implement sustainable development. The implications of the food waste issue go beyond economy, with grave social human societies [3]. Food spoilage is virtually destroying the livelihood of the planet and vital resources of humanity. The far-reaching consequences of food spoilage establish it as an especially obvious example of the profligacy of human societies and deviation from the criteria of sustainable development [3] and environmental consequences also significantly affecting the sustainability of the development of the majority of food waste in developing and underdeveloped countries stems from poor management and lack of proper technology in the distribution and retail sectors. In the case of developed countries, however, food waste is mostly caused by an increasingly prevalent consumerist attitude. It is estimated that the production of this amount of wasted food each year involves the cultivation of 1.98 million km^2^ of agricultural land, 173 billion m^3^ of fresh water, and emission of up to 5,600 million m^3^ of greenhouse gases (GHGs) [4, 5]. As Fig 2 shows, meat and its byproducts rank third among the largest sources of food waste in the world.

In the food industry, the application of hygienic practices is considered critically important, especially when it comes to processed food factories. Therefore, the water used in industrial food production processes should be as clean as drinking water. In recent years, the food industry has become one of the leading industries in Iran and has a significant role in the overall development of the country. Food companies have made tremendous efforts to promote their respective industry in order to not only motivate meat and chicken producers to expand their business, but also to compensate for the high price of meat by changing the people’s general food culture toward less expensive options. There are currently about 170 meat factories in Iran [6, 7]. As some of the oldest and most widely-consumed ready meals in the world, sausages and lunch meats also have a special place in the diet of average Iranians. According to statistics, the average per-capita consumption of these meat products in Iran was 1.5 kg per year between 2012 and 2021. However, in recent years, the figure has exceeded 5.5 kg and over 500 thousand tons of sausages and lunch meats are consumed annually in the country [7]. Meanwhile, the per-capita consumption of the products is 60 kg in European countries and 70 kg in the United States [3]. On the other hand, food waste is a serious problem in Iran. The initiatives to reduce or control food waste have thus become important in ensuring the food security of the nation. According to a report by the United Nations’ Food and Agriculture Organization (FAO), the annual food waste in Iran amounts to 35 million tons, whereas the European Union, comprising 27 member states and a total population of nearly 450 million, generates only 90 million tons of food waste per year [8]. According to research, 15 billion dollars’ worth of food is wasted each year in Iran, most of which caused by spoilage [9]. For decades, the industrial sectors of developed countries have been working on ways of controlling food waste for years through the use of increasingly efficient, high-tech equipment in the production and recycling processes. In addition to incurring considerable costs, this crisis means that a great deal of the water used in the production of foods is wasted, as well. According to reports, nearly 7 billion m^3^ of water is consumed each year for drinking and industrial purposes in Iran [6]. SC management refers to a global strategy to achieve organizational competitiveness. Designing a SC involves strategic decisions (e.g. facility location) that determine the configuration of the network. Considering the sustainability criteria can mitigate the adverse environmental effects and enhance the positive social impact of SCs, benefits that can explain the recent growth in the number of academic studies on sustainable SCs. Demand also plays a crucial role in the design of any SC model and, since price and demand are closely related, sales prices affect the quantity of demand [10–12]. Customers’ purchase decisions are strongly influenced by prices. Most food products have a limited lifetime. That is, the deterioration rate of the products gradually increases and reaches 100% by their expiration date [10]. When customers purchase perishable products, they tend to choose those with longer expiration dates to ensure a longer storage time [11]. Since quality and freshness are key characteristics of foods, direct or indirect shipment in a food SC can also have a significant impact on on-time deliveries [13, 14]. While research appears to have mainly been concentrated on the economic aspects, few studies have focused on the environmental and social consequences of food SCs [15].

Since sustainable development has become a vital component of today’s SCs, there is a growing need for techniques of developing mathematical models of SCs that integrate social, environmental, and economic objectives. Most of the research on perishable product SCs has revolved around the inventory control in single- and bi-level chains, often with economic goals. Products with fixed expiration dates (a.k.a. shelf lives) are often assigned lower spoilage and quality-degradation rates. In the present study, we propose a multi-objective model to design a SC of perishable food products with four components: a supplier, a PC, DCs, and retailers, all of which should comply with the three dimensions of sustainability. In this study, the two environmental indicators include the treatment of the wastewater generated by the PC sector and the emission of the Co_2_ generated by facility construction and transportation vehicles. In the social dimension, job creation and regional development are the key factors. When it comes to determining the optimal price and the optimal time to place orders for products to reduce the waste caused by spoilage in the distribution centers, two main factors are considered: the spoilage rate (which depends on the expiration date) and demand (which depends on price). To maintain the freshness and quality of the product and to make the chain more responsive, direct shipment from the PC is considered for the retails within its coverage radius. Finally, the presented model is evaluated in a real meat products industry in Iran with three products: sausage, kielbasa, and hamburger.

The remainder of this paper is organized as follows. The literature on the subject is reviewed in Section 2. The problem is described in Section 3. The multi-objective mixed-integer non-linear programming model developed through the course of this research is elaborated in Section 4. The case study is presented in Section 5. Our solution approach, which is based on preemptive fuzzy goal programming (PFGP), is introduced in Section 6. The computational results are detailed in Section 7. A brief conclusion and recommendations for future research are provided in Section 8. Lastly, the managerial implications derived from our findings are outlined in Section 9.

## 2. Literature review

Managing the SCs of perishable goods with short lifecycles has always represented a challenge and researchers have scrutinized the different aspects of this problem and proposed various solutions. Mosallanezhad et al. [16] classified foodstuffs into two groups of perishable (aquaculture products, meats, fruits, etc.) and non-perishable (canned, pickled, dried, etc.) products, and worked on minimizing the total cost of a shrimp SC. Several studies have investigated food SCs. Mohammad-Musavi and Bozorgi-Amiri [17] and Rabbani et al. [18] studied the product quality and freshness considering economic objectives, while Mogale et al. [19] studied the Indian public distribution system’s transportation-allocation SC of edible seeds, such as cereal grains, to minimize the total cost. Onggo et al. [20] considered a SC of perishable agro-food products to minimize the costs while facing stochastic customer demand. Through a case study, Tabrizi et al. [21] examined a warm-water fish farm to maximize the profit of the SC components. De et al. [22] presented a model for a Norwegian salmon SC that addressed environmental concerns and aimed to minimize the fuel cost and Co_2_ emissions. Bortolini et al. [23] replaced non-renewable energy sources (fossil fuels) by renewable ones (solar, wind, biomass, etc.) in the food SC. As for comprehensive approaches specifically developed for food SCs, Gomes et al. [24] proposed a method to maximize the value of economic and environmental indices. Accorsi et al. [25] created a tomato SC model on the basis of three main linear functions: 1) total SC cost, 2) GHG emissions, and 3) energy requirements. Some studies have surveyed meat SCs with the goal of minimizing the costs and environmental impacts [26], especially Co_2_ emission, social consequences (delivery rate) [27] and maximum capacity utilization [28]. Rahbari et al. [29] presented a novel multi-period location-inventory-routing model for a red meat SC with various vehicle fleet and logistics decisions. Liu et al. [30] conducted a similar study for a SC of perishable products considering the pillars of sustainability. Alidadi Talkhestani et al. [31] studied a location-inventory model for a supply chain with perishable products with price-dependent demand. Jouzdani and Govindan [32] developed a model for perishable products to optimize costs, energy consumption, and traffic congestion. Amorim et al. [33] presented a processed food SC to optimize the supplier selection process and minimize the risks associated with product delivery. The same authors would later (2016) expand on their model by considering the products’ spoilage and lifecycles [34]. A number of studies have attempted to minimize the economic and environmental objectives and maximize job creation [14, 35, 36], work on regional development [37], or minimize injury-caused unemployment days [36]. Varsei and Polyakovskiy [38] presented a model for a winery considering the three sustainability criteria. Some authors have studied the economic objectives, impact of GHGs [39], and service levels, while others have taken customer health as an environmental and social factor [40]. Tavakoli-Moghaddam et al. [41] utilized technology to develop sustainable reverse SCs for perishable items with the goal of maximizing the profit and customer satisfaction, as well as minimizing the costs and adverse environmental impacts. Mogale et al. [42] developed a mathematical model considering procurement, transportation, inventory, and location-related issues and assessed its performance in terms of the sustainability dimensions. Rahbari et al. [43] developed a novel mathematical model for a four level four level multi-product multi-period canned food SC to minimize costs considering strategic and tactical decisions.

Food deterioration is now a serious food-industry challenge in all parts of the world because the short shelf life of perishable foods such as meats and dairies cause their demand rate to gradually decline to zero as the products’ expiration date approaches. Our review of the body of research on this subject points to a clear relationship between price and demand, where each sales price appears associated with a certain level of demand. Pricing policies are meant to increase the overall profitability of SCs, particularly when it comes to the distribution network. Many studies have proposed inventory models of price-dependent demand for perishable items. Yang et al. [44] investigated a closed-loop logistics system with price-sensitive demand. Wang et al. [45] considered expiration date-dependent deterioration rates and exponential price-dependent demands. Tayal et al. [46] examined price-dependent demands and deterioration rates. Adeinat and Ventura [47] proposed a model to find the optimal sales price and reimbursement policy for a particular type of product. Wu et al. [48] presented a hybrid economic-order quantity model for perishable products with expiration date-dependent deterioration rates and a trapezoidal price-time-dependent demand function. In some papers, the demand function is related to the sales price by linear decreasing functions and continuous deterioration rates [49, 50] or expiration date-dependent deterioration rates [10, 51–54]. Aiming at maximizing the profit of a three-level SC, Kianfarˈs model sets the demand function to depend on the price and scope of advertisements [55]. Agrawal and Yadav [56] studied SCs that considered price-dependent demands to simultaneously define the production, inventory, and pricing decisions. Macias-Lopez et al. [57] developed an inventory model where both the physical and freshness depreciations were constrained. The demand for perishable products is a multivariate function of freshness, current inventory level, and price. Rahman et al. [11] demonstrated the optimal strategy of an inventory system for perishable goods with hybrid demand that depended on the sales price and stock under a known fixed-ratio partial backlogging. Mishra et al. [58] presented an inventory model for perishable products to maximize profit. The model had four Production-rate levels and demand dependent on rebate value. In this model, optimal replenishment, quantity of order, and selling price were prioritized.

Since the quality and freshness of food products are essential, in the specific case of food SCs, the choice between direct and indirect shipment methods may highly affect the state in which a given product reaches consumers. In the majority of the studies reviewed in this research, food products are distributed through DCs or wholesalers; however, some scholars have also assessed direct PC-to-retailer shipment methods. In multi-level SCs, although the choice between direct and indirect shipments is mostly made with the purpose of minimizing the shipping costs, other objectives such as maximizing capacity utilization [59] or total profit [14] are also involved. Goodarzi and Zegordi [60] compared direct supplier-to-PC/DC shipments and indirect shipments via terminals or cross-docks. Pishvaee and Rabbani [61] and Salehi et al. [59] investigated direct shipment cases as opposed to when this was not possible. Some papers addressed direct product shipment from the production center to the end-consumer through regular transportation and indirect shipment through DCs [14, 43, 62]. In integrated production-inventory-transportation decisions [63] and inventory-routing problems [13], both direct shipment (from supplier/PC to customer) and indirect shipment (from supplier/PC to DCs and then to retailers/customers) methods have been used to deliver a single or multiple products.

**Fig 3:**
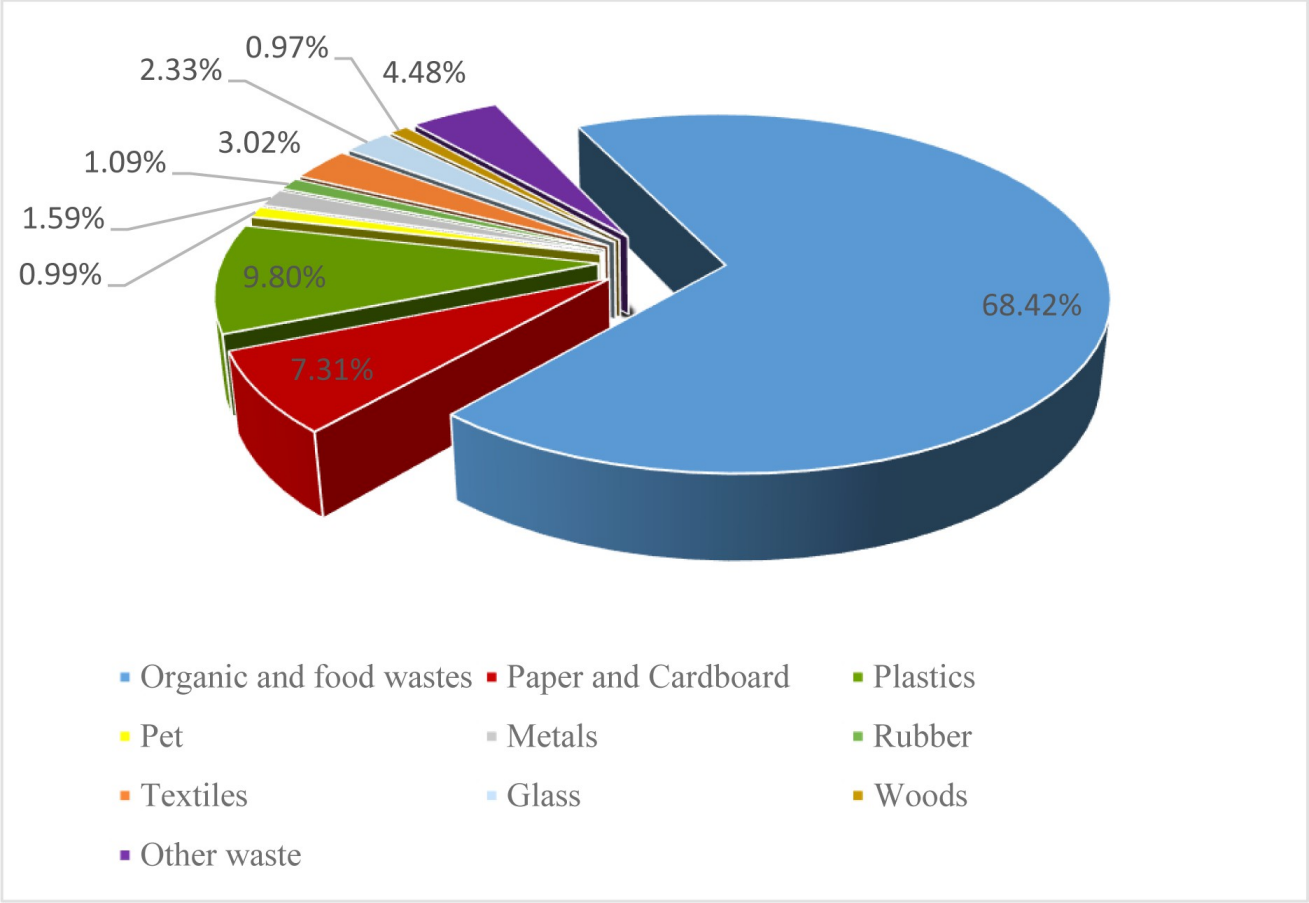
Average composition of municipal solid waste in Iran (2009–2019) [8].

As mentioned earlier, the tendency toward consuming ready meals and processed products has increased among the world population and the food consumption patterns around the world have undergone a transformation in parallel with the incessant changes in lifestyles and occupations. Current projections indicate that the global demand for processed meat products will continue to grow in the foreseeable future. It is estimated that more than 40% of the food produced in the world is either thrown away or wasted. In addition to the economic aspect, such wastefulness leads to considerable social and environmental damages. The consequences of food waste are symptomatic of the deviation of governments and societies from the principles of sustainable development. The majority of food waste in developing and underdeveloped countries is due to mismanagement and poor operating practices in DCs and retail stores. On the other end of the spectrum, a pervasive consumerist attitude is thought to be responsible for most of the food waste in developed countries. The ration of the food wasted in the Middle East is estimated to be 18 to 26%. Food waste is also a serious problem in Iran (Fig 3). There is little doubt that the reduction and control of food waste are critical to the country’s food security. In addition, industries in developed countries have been exploring ways of preventing or controlling food waste for years and already use efficient high-tech equipment in production and recycling procedures to minimize the amount of wasted food. In Iran, most of the waste caused by spoilage occurs in DCs and retail stores. The decisive factor in this regard is perhaps the general consumption pattern of population, although eating habits, buying more food than currently needed, pricing and production mechanisms, and poor storage or packaging are all important factors aggravating this issue. An effective way of controlling the cited factors is demand-based pricing and determining optimal order quantities in the DC and storage centers to ensure better inventory management [64].

**Fig 4:**
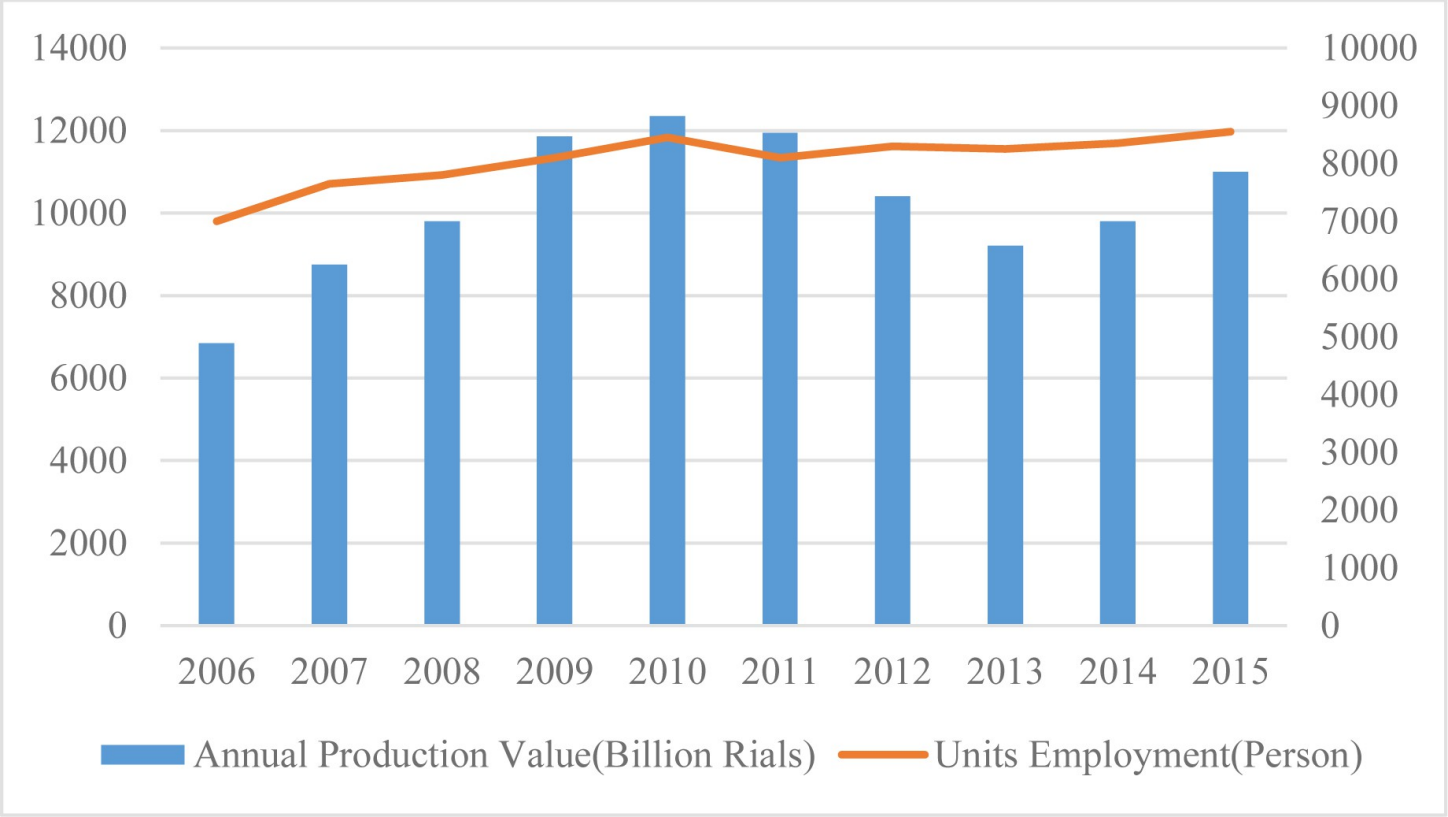
Annual production value and employment by the meat industry [6].

According to statistics, 16% of the wastewater in Iran is generated by the industrial sector. The food industry disposes of 50% of the water it consumes in the form of wastewater, which can then be used for non-drinking purposes such as irrigation and cleaning. In the food industry in general, and particularly when it comes to processed food products, compliance with hygienic standards and general cleanliness in the manufacturing plants are important. Therefore, the water used in the food industry is treated with the same sensitivity as drinking water. The usual purification methods can be applied to ensure of the water’s safety and quality. The meat industry has traditionally had a significant role in Iran from a socioeconomic standpoint. Fig 4 shows the number of jobs created based on the production volume of the products in this industry.

A thorough review of the relevant studies in Section 2, summarized in Table 1, outlines the gaps in the literature as follows:

1. Despite the importance of sustainability in SCs, the three indicators of sustainability have not been widely incorporated by researchers in the design of perishable food product SCs.
2. Few researchers have considered the spoilage rate according to the expiration date or the perishable nature of food products in multi-level SCs.
3. Few Researches have paid attention to the wastewater treatment in the PC, which is important in the food industry, as an environmental indicator and regional development as a social indicator.

To bridge the above-mentioned gaps in the literature, we propose a nonlinear multi-objective multi-product model considering the three indicators of sustainability to design a four-level perishable food product SC with price-dependent demand. The four components of the SC include a supplier, a PC, DCs, and retailers. The quality degradation rate depends on the expiration date, which is therefore considered as another element of the model. Wastewater treatment in the PC sector, in addition to Co_2_ emissions caused by the construction of PC, DCs, and transportation facilities in the SC are regarded as the environmental indicators. The social dimension is represented by the number of jobs created and the extent of regional development. In the end, the model is applied to a case study of a processed meat company with three groups of products: sausages, kielbasa, and hamburgers.

**Table 1:** Reviewed related literature.

**Reference**	**Model**										**Sustainability**								**Modelling Approaches**	**Solution Methods**	**Application**
	Network echelons	Demand Function		Pricing	Cycle Length	Delivery Modes		Deterioration Function		Multiple Product	Economic		Environmental			Social					
		Constant	Non-Constant			Direct	In-Direct	Constant	Non-Constant		Cost Minimization	Profit Maximization	Co_2_ Emission	Wastewater Treatment	Others	Job Creation	Regional Development	Others			
**Mohammed and Wang (2017)**	S,P,R	√									√		√					√	FMOP	Max-Min	Meat
**Zhalechian et al. (2017)**	H										√					√	√	√	MINLP	EA	General
**Tavakoli Moghaddam et al. (2018)**	P,DC,R	√								√	√	√	√		√			√	LP	GAMS	Processed Food
**Khan et al. (2019)**	R		√	√	√				√			√							NLP	Mathematically	Perishable Products
**Mohammadi et al. (2020)**	S,P,DC,R	√				√	√	√		√		√	√	√		√			MILP	Augmented ɛ-constraint	Processed Food
**Manteghi et al. (2020)**	S,P		√	√						√	√				√			√	NLP	Game Theory	Food
**Agrawal and Yadav(2020)**	P,R		√	√								√							MINLP	GA and TLBO	General
**Jouzdani and Govindan(2020)**	DC,R	√								√	√				√			√	MINP	GP	Daily Products
**Gholami-Zanjani et al. (2020)**	P,DC,C	√								√	√								MILP	LWTM	Food
**Azizi and Hu (2020)**	S,DC,C	√				√	√			√	√								NP-Hard	DM,OM,BD	General
**Mohebalizadehgashti et al. (2020)**	S,P,R,C	√		√						√	√		√					√	MILP	Augmented ɛ-constraint	Meat
**Liu et al. (2021)**	P,DC,R	√								√	√		√					√	MOP	YALMIP	Perishable Products
**Rahman et al. (2021)**	R		√		√			√				√							NLMP	Mathematically	Perishable Products
**Huang et al. (2021)**	R		√	√				√				√							NLP	Mathematically	Perishable Food
**Kalantari and Hosseininezhad (2021)**	S,P,DC,C	√								√	√		√			√			MOP	CE	Food
**Rahbari et al. (2022)**	S,P,DC,R	√									√								MILP	GAMS	Red meat
**Alidadi Talkhestani et al. (2022)**	P,DC,R,C		√	√		√	√			√		√							MINLP	GAMS	Perishable Products
**Choudhury et al. (2022)**	P,R		√	√	√				√			√							NLP	New Algorithm	Perishable Products
**Jani et al. (2023)**	S,R		√	√	√				√			√							NLP	AR	General
**Mishra et al. (2023)**	R		√	√	√			√				√							NLP	Mathematically	Perishable Products
**Mahato et al. (2023)**	R		√	√	√				√			√							NLP	Mathematically	General
**Mogale et al. (2023)**	F,PC,BS,FS,RS, FPS	√									√		√			√		√	MINLP	MOGLNPSO and MOPSO	Agri-food
**Rahbari et al. (2023)**	S,P,DC,R	√				√	√			√	√								MILP	GAMS	Canned food
***This Research***	***S*,*P*,*DC*,*R***		***√***	***√***	***√***	***√***	***√***		***√***	***√***	***√***	***√***	***√***	***√***		***√***	***√***		***MINLP***	***PFGP***	***Processed Meat Food***

S = Supplier, P = Production center, DC = Distribution center, R = Retailer, C = Customer zone, H = Hub, FMOP = Fuzzy Multi-Objective Programming, NLP = Non-Linear Programming, LP = Linear Programming, MILP = Mix Integer Linear Programming, MINLP = Mix Integer Non-Linear Programming, NLMP = Non-Linear Maximization Problem, MOMMLRP = Multi-Objective Many-to-Many Location-Routing Problem, MOP = Multi-Objective Programming, GA = Genetic Algorithm, TLBO = Teaching-Learning- Based Optimization, LWTM = Lexicographic Weighted Tchebycheff Method, DM = Deterministic Mode, OM = Opportunistic Mode, BD = Benders Decomposition algorithm, CE = Cross Entropy algorithm, EA = Evolutionary algorithm, AR = Algorithm Rule, MOGLNPSO = Multi-Objective Particle Swarm Optimization Algorithm with Gbest, Lbest and Nbest, MOPSO = Multi-Objective Particle Swarm Optimization, F = Farmers, PC = Procurement Centers, BS = Base Silos, FS = Field Silos, RS = Regional Silos, FPS = Fair Price Shops

## 3. Problem description

**Fig 5:**
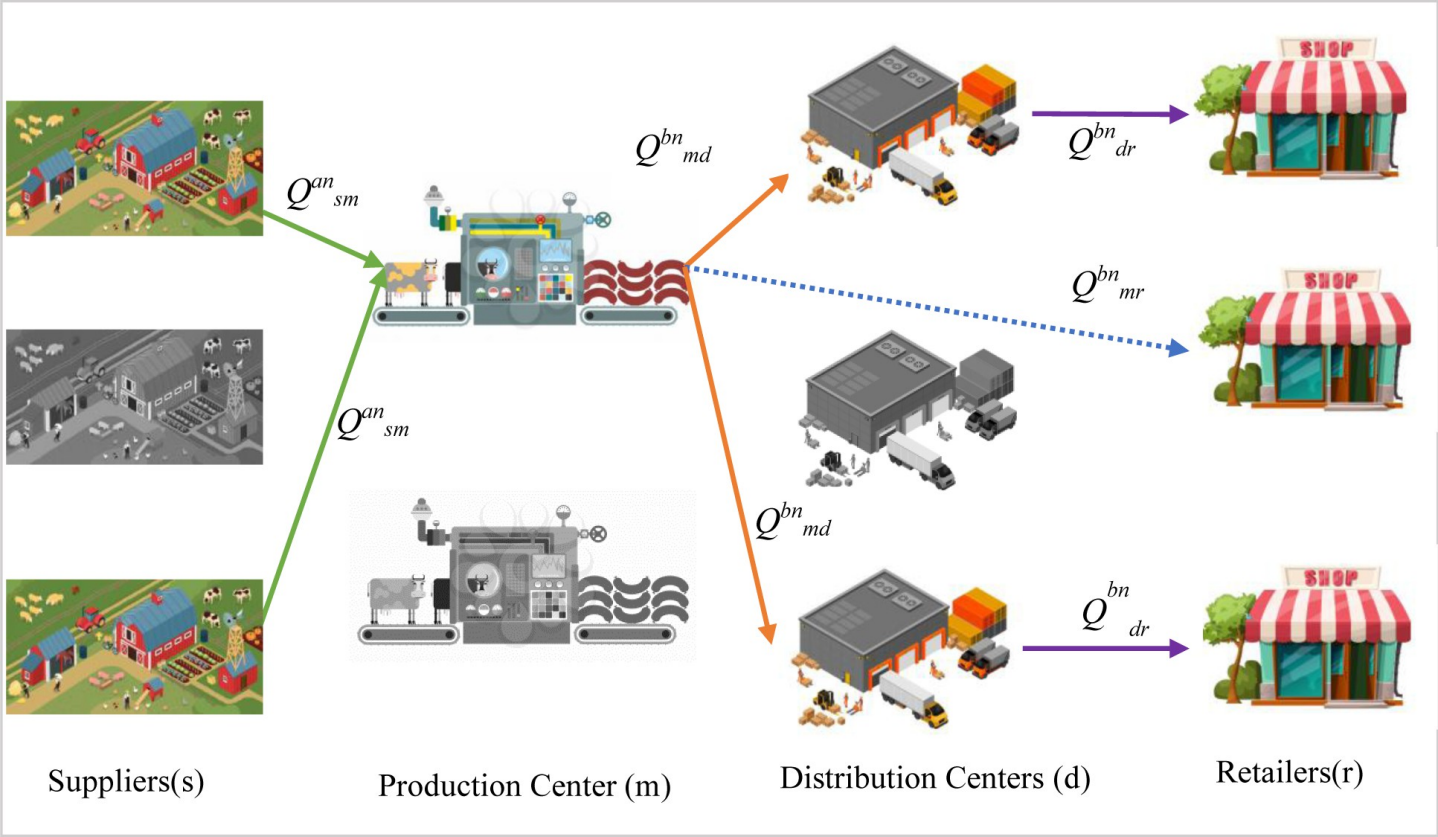
Structure of the present study’s proposed SC network.

A SC is a product-delivery cycle which typically consists of PC, DCs, and other components. In this study, we design a SC with multiple PCs, suppliers, DCs, and retailers. The locations of the suppliers and retailers are predetermined, while the DCs and PCs are set up in pre-existing candidate locations. The raw materials required by the PC are supplied by various suppliers. The SC involves multiple perishable products with fixed shelf lives (a.k.a. expiration dates) and mechanized delivery vehicles with different capacities handle the transportation of the raw materials and finished products. The products are either shipped directly from the PCs to the retailers, where the demand is known and fixed, or indirectly through DCs where the demand depends on the sales price. The products’ deterioration rate depends on their expiration date, while the replenishment rate of the DCs is limited and should be done immediately after the last item has been sold. The proposed model locates the PC and DCs to maximize the profit by minimizing the costs of setting up the PCs and DCs, purchasing raw materials, placing orders, purchasing and holding products in the DCs, transportation throughout the entire network, wastewater treatment in PC; and the Co_2_ emitted by the construction of the production and distribution centers as well as by the transportation vehicles traveling among the SC nodes. The model also maximizes the employment and regional development indices by setting the PC and DCs as social indicators. Fig 5 shows the structure of the proposed SC.

The main underlying assumptions of the model are as follows:

1. Setting up the PC and DCs has a fixed cost and a variable cost, such that the latter is a coefficient of the former.
2. The retailers’ demand must be fully satisfied.
3. The retailers’ demand is predetermined and fixed when direct shipment is involved; however, for indirect shipments, the demand depends linearly on the sales price: $D_{r}^{b}=a_{r}^{b}-b_{b}{Pr}_{dr}^{b}$ where *a*^*b*^_*r*_ is the retailer’s demand when the sales price is zero (*Pr*^*b*^_*dr*_ = 0) and *b*_*b*_ is the demand reduction coefficient when the price is larger than zero: *a*^*b*^_*r*_*—b*_*b*_*Pr*^*b*^_*dr*_ *> 0*.
4. Since the products are perishable and have an expiration date, their deterioration rate *θ*^*b*^(*t*) is defined as follows [10]:
$$\theta^{b}(t)=\frac{1}{1+{exp}_{b}-t}.0\leq t\leq T_{d}^{b}\leq {exp}_{b}$$ (1)
  where the deterioration rate is an incremental function over time and depends on the product’s expiration date (*exp*_*b*_). Notably, the replenishment cycle length (*T*^*b*^_*d*_) cannot exceed the maximum length of the lifecycle because the product cannot be sold after its expiration date.
5. The transportation fleet is mechanized and consists of vehicles with different capacities.
6. The quantity of Co_2_ emitted by the transportation vehicles depends on the distance and number of deliveries made between the nodes.

## 4. Mathematical model

We will introduce the sets, parameters, decision variables and the mathematical model for the previously problem, in this section.

### 4.1. Notations

**Table:** 

***Sets*:**		
*s*	The set of supplier(*s = 1*,*…*,*S*)	
*m*	The set of PC(*m = 1*,*…*,*M*)	
*d*	The set of distribution center(*d = 1*,*…*,*D*)	
*r*	The set of retailer(*r = 1*,*…*,*R*)	
*a*	The set of raw material(*a = 1*,*…*,*A*)	
*p*	The set of final product(*b = 1*,*…*,*B*)	
*n*	The set of transportation mode(*n = 1*,..,*N*)	
***Parameters***:		
*D* ^*b*^_*r*_	The demand quantity for product *b* by retailer *r*;	
*a* ^*b*^_*r*_	Constant parameter related to the demand function of the retailer *r* for product *b* in indirect shipping(*a* >0);	
*b*_*b*_	Price reduction factor for product *b* in the demand function(*b* >0);	
*Pr* ^*b*^_*mr*_	Selling price per unit of the PC *m* to retailer *r* for product *b*;	
*μ* ^*a*^_*b*_	Consumption coefficient of raw material *a* in product *b*;	
*FP*_*m*_	PC opening fixed cost in the candidate location *m*;	
*α*	PC opening variable cost coefficient in the candidate location *m*;	
*FD*_*d*_	DC opening fixed cost in the candidate location *d*;	
*β*	DC opening variable cost coefficient in the candidate location *d*;	
*C* ^*a*^_*s*_	Ordering cost raw material *a* from supplier *s*;	
*CS* ^*a*^_s_	Capacity of supplier *s* for raw material *a*;	
*CP* ^*b*^_*m*_	Capacity of potential PC *m* for product *b*;	
*CD* ^*b*^_*d*_	Capacity of DC *d* for product *b*;	
*CT*_*n*_	Capacity of transportation mode *n*;	
*CW*_*m*_	Capacity of wastewater facility in PC *m*;	
*exp*_*b*_	Maximum shelf-life (Expire date) for product *b*;	
*K* ^*b*^_*d*_	Ordering cost of product *b* in the DC *d*;	
*C* ^*b*^_*md*_	Purchasing cost per unit product *b* by the DC *d* from PC *m*;	
*TC* ^*an*^_*sm*_	Transportation cost between supplier *s* and PC *m* for raw material *a* by mode *n*;	
*TC* ^*n*^_*md*_	Transportation cost between PC *m* and DC *d* by mode *n*;	
*TC* ^*n*^_*mr*_	Transportation cost between PC *m* and retailer *r* by mode *n*;	
*h* ^*b*^_*d*_	Holding cost per unit product *b* in DC *d*;	
*EF*_*m*_	Purchasing cost per unit of fresh water in PC *m*	
*EW*_*m*_	Filtration cost per unit of wastewater in PC *m*;	
*Η*_*m*_	Penalty cost per unit of non-filtration wastewater in PC *m*;	
*Ω*	Maximum passable level dumping non-filtration wastewater;	
*ρ*_*m*_	Wastewater filtration rate in PC *m*;	
*π* ^*a*^_*m*_	Water quantity needed in PC *m* to preparation each unit of the raw material *a*;	
*b* ^*a*^_*m*_	Processing cost in PC *m* for each unit of raw material *a*;	
*d* ^*a*^_*sm*_	Distance between supplier *s* to PC *m* for raw material *a*;	
*d*_*md*_	Distance between PC *m* to DC *d*;	
*d*_*mr*_	Distance between PC *m* to retailer *r*;	
*d*_*dr*_	Distance between DC *d* to retailer *r*;	
*JCUP*_*m*_	Number of created job opportunities in open PC *m*;	
*JCUD*_*d*_	Number of created job opportunities in open DC *d*;	
*up*_*m*_	Unemployment rate at PC location *m;*	
*up*_*d*_	Unemployment rate at DC location *d*;	
*EV*_*m*_	Economic value in the region of PC *m*;	
*EV*_*d*_	Economic value in the region of DC *d*;	
*re*_*m*_	Regional development coefficient in the location of PC *m*;	
*re*_*d*_	Regional development coefficient in the location of DC *d*;	
*Co*_*2m*_	Co_2_ emission in gram of establishing PC *m*;	
*Co*_*2d*_	Co_2_ emission in gram of Establishing DC *d*;	
*Co*_*2n*_	Per kilometer emission rate of Co_2_ gas in gram form transportation mode *n*;	
*M*	Big number	
***Variables***:		
*Q* ^*an*^_*sm*_	Quantity of raw material *a* shipped by transportation mode *n* from supplier *s* to PC *m;*	
*Q* ^*bn*^_*md*_	Quantity of product *b* shipped by transportation mode *n* from PC *m* to DC *d*;	
*Q* ^*bn*^_*mr*_	Quantity of product *b* shipped by transportation mode *n* from PC *m* to retailer *r*;	
*Q* ^*bn*^_*dr*_	Quantity of product *b* shipped by transportation mode *n* from DC *d* to retailer *r*;	
*Pr* ^*b*^_*dr*_	Salling price per unit of product *b* in indirect shipment from DC *d* to retailer *r*;	
*T* ^*b*^_*d*_	Replenishment cycle length in DC *d* for product *b*.	
*P*_*m*_	$\left\{ \begin{array}{l} 1\;if\;PC\;is\;established\;in\;location\;m\\ 0\;otherwise \end{array}\right.$	
*DC*_*d*_	$\left\{ \begin{array}{l} 1\;if\;DC\;is\;established\;in\;location\;d\\ 0\;otherwise \end{array}\right.$	
*Tr*_*n*_	1 $\left\{ \begin{array}{l} \;if\;transportation\;mode\;n\;is\;selected\\ 0\;otherwise \end{array}\right.$	

### 4.2. Objective function


$$\mathrm{Max}\;profit=[\sum_{b}\sum_{m}\sum_{r}\sum_{n}{Pr}_{mr}^{b}∙Q_{mr}^{bn}+\sum_{b}\sum_{d}\sum_{r}\sum_{n}{Pr}_{dr}^{b}∙Q_{dr}^{bn}]-[\sum_{a}\sum_{s}\sum_{m}\sum_{n}C_{s}^{a}∙Q_{sm}^{an}+\sum_{a}\sum_{s}\sum_{m}\sum_{n}\lceil \frac{Q_{sm}^{an}}{{CT}_{n}}\rceil {TC}_{sm}^{an}+\sum_{b}\sum_{m}\sum_{d}\sum_{n}\lceil \frac{Q_{md}^{bn}}{{CT}_{n}}\rceil {TC}_{md}^{n}+\sum_{b}\sum_{m}\sum_{r}\sum_{n}\lceil \frac{Q_{mr}^{bn}}{{CT}_{n}}\rceil {TC}_{mr}^{n}+\sum_{b}\sum_{d}\sum_{r}\sum_{n}\lceil \frac{Q_{dr}^{bn}}{{CT}_{n}}\rceil {TC}_{dr}^{n}+\sum_{b}\sum_{d}K_{d}^{b}∙{DC}_{d}+\sum_{b}\sum_{m}\sum_{d}\sum_{n}c_{md}^{b}∙Q_{md}^{bn}+\sum_{b}\sum_{r}\sum_{m}\sum_{d}h_{d}^{b}∙Q_{dr}^{bn}[\frac{{\left(1+{exp}_{b}\right)}^{2}}{2}\ln\left(\frac{1+{exp}_{b}}{1+{exp}_{b}-T_{d}^{b}}\right)+\frac{{T_{d}^{b}}^{2}}{4}-\frac{(1+{exp}_{b})T_{d}^{b}}{2}]+\sum_{a}\sum_{s}\sum_{m}\sum_{n}(\rho_{m}∙\pi_{m}^{a}∙{EW}_{m}+(1-\rho_{m})\pi_{m}^{a}∙\eta_{m}+\pi_{m}^{a}∙{EF}_{m})Q_{sm}^{an}]$$ (2)

In the model proposed in this research, the first objective function (2) minimizes the fixed and variable setup costs of the PC and DCs and the second objective function (3) maximizes the profit. Since the construction cost is one of the strategic and long-term goals, it makes the profit function, which is tactical and short-term, a negative value. The reason is that the model is defined as single-period to determine the length of replenishment periods based on the decay rate according to the expiration date. Therefore, we have considered the two functions, which are of the same type but have different time horizons, separately. The first part of the second objective function considers the SC revenue with direct PC-retailer shipment while the demand and sales price are known. With the indirect PC-DC-retailer shipment, however, the demand is price-dependent. In the second part, the tactical SC costs include those incurred by procuring raw materials from the suppliers, transportation among the SC nodes, order placement costs in the DCs, purchasing the products from PCs by DCs, holding the products at DCs, treating the wastewater in PCs, and the decay of products in DCs.


$$\mathrm{Max}\;social=\left(\sum_{m}{JCUP}_{m}∙{up}_{m}∙P_{m}+\sum_{d}{JCUD}_{d}∙{up}_{d}∙{DC}_{d}\right)+\left(\sum_{m}{EV}_{m}(1-{re}_{m})P_{m}+\sum_{d}{EV}_{d}(1-{re}_{d}){DC}_{d}\right)$$ (3)

The third objective function (4) maximizes two social indices: job creation and economic development of the regions in which the PCs and DCs are established according to the region’s current unemployment rate and human development index.


$$\mathrm{Min}\;environmental=\sum_{m}{{Co}_{2}}_{m}∙P_{m}+\sum_{d}{{Co}_{2}}_{d}∙{DC}_{d}+\sum_{a}\sum_{s}\sum_{m}\sum_{n}{{Co}_{2}}_{n}\lceil \frac{Q_{sm}^{an}}{{CT}_{n}}\rceil d_{sm}^{a}+\sum_{b}\sum_{m}\sum_{d}\sum_{n}{{Co}_{2}}_{n}\lceil \frac{Q_{md}^{bn}}{{CT}_{n}}\rceil d_{md}+\sum_{b}\sum_{m}\sum_{r}\sum_{n}{{Co}_{2}}_{n}\lceil \frac{Q_{mr}^{bn}}{{CT}_{n}}\rceil d_{mr}+\sum_{b}\sum_{d}\sum_{r}\sum_{n}{{Co}_{2}}_{n}\lceil \frac{Q_{dr}^{bn}}{{CT}_{n}}\rceil d_{dr}$$ (4)

Lastly, the fourth objective function (5) minimizes the Co_2_ emitted as a result of setting up the PCs and DCs and by the transportation vehicles traveling among the SC nodes.

### 4.3. Constraints

Constraints (6–10) place restrictions on the process of setting up the facilities, the quantity of raw materials delivered from the suppliers to the PCs, and shipping the products from the PCs to DCs or retailers and from DCs to retailers if the PCs and DCs have already been set up.


$$Q_{sm}^{an}\leq M∙P_{m}\;\forall a,s,m,n$$ (5)

$$Q_{md}^{bn}\leq M∙P_{m}\;\forall b,m,d,n$$ (6)

$$Q_{md}^{bn}\leq M∙{DC}_{d}\;\forall b,m,d,n$$ (7)

$$Q_{mr}^{bn}\leq M∙P_{m}\;\forall b,m,r,n$$ (8)

$$Q_{dr}^{bn}\leq M∙{DC}_{d}\;\forall b,d,r,n$$ (9)

Constraints (11–13) state the maximum capacities of the suppliers, PCs, and DCs, respectively.


$$\sum_{m}\sum_{n}Q_{sm}^{an}\leq {CS}_{s}^{a}\;\forall a,s$$ (10)

$$\sum_{r}\sum_{n}Q_{mr}^{bn}+\sum_{d}\sum_{n}Q_{md}^{bn}\leq {CP}_{m}^{b}∙P_{m}\;\forall b,m$$ (11)

$$\sum_{m}\sum_{n}Q_{md}^{bn}\leq {CD}_{d}^{b}∙{DC}_{d}\;\forall b,d$$ (12)

Constraints (14–17) express the capacity limitations of the transportation vehicles.


$$Q_{sm}^{an}\leq {Ct}_{n}∙{Tr}_{n}\;\forall a,s,m,n$$ (13)

$$\sum_{b}Q_{md}^{bn}\leq {Ct}_{n}∙{Tr}_{n}\;\forall m,d,n$$ (14)

$$\sum_{b}Q_{mr}^{bn}\leq {Ct}_{n}∙{Tr}_{n}\;\forall m,r,n$$ (15)

$$\sum_{b}Q_{dr}^{bn}\leq {Ct}_{n}∙{Tr}_{n}\;\forall d,r,n$$ (16)

Constraints (18) and (19) ensure a balanced product flow in the PCs and DCs, respectively.


$$\sum_{s}\sum_{n}Q_{sm}^{an}=\mu_{b}^{a}(\sum_{r}\sum_{n}Q_{mr}^{bn}+\sum_{d}\sum_{n}Q_{md}^{bn})\;\forall a,b,m$$ (17)

$$\sum_{m}\sum_{n}Q_{md}^{bn}=\sum_{r}Q_{dr}^{bn}(1+{exp}_{b})\ln\left(\frac{1+{exp}_{b}}{1+{exp}_{b}-T_{d}^{b}}\right)\;\forall b,d$$ (18)

Constraint (20) ensures that the retailers’ demand is met either by direct or indirect shipment of products. Constraints (21) and (22) indicate the retailer’s demands under direct and indirect shipment conditions, respectively.


$$\sum_{m}\sum_{n}x_{mr}^{bn}+\sum_{d}\sum_{n}y_{dr}^{bn}=1\;\forall b,r$$ (19)

$$\sum_{m}\sum_{n}Q_{mr}^{bn}=\sum_{m}\sum_{n}{D_{r}^{b}.x}_{mr}^{bn}\;\forall b,r$$ (20)

$$\sum_{d}\sum_{n}Q_{dr}^{bn}=\sum_{n}\sum_{d}\left(a_{r}^{b}-b_{b}.{Pr}_{dr}^{b}\right){.y}_{dr}^{bn}\;\forall b,r$$ (21)

Constraint (23) defines the capacity limitations of the wastewater treatment facilities. Constraint (24) ensures that the amount of wastewater entering the environment does not exceed the permissible limit.


$$\sum_{a}\sum_{s}\sum_{n}\rho_{m}.\pi_{s}^{a}.Q_{sm}^{an}\leq P_{m}.{CW}_{m}\;\forall m$$ (22)

$$\sum_{a}\sum_{s}\sum_{n}(1-\rho_{m})\pi_{m}^{a}.Q_{sm}^{an}\leq \Omega \;\forall m$$ (23)

Constraints (25–27) indicate the replenishment time interval based on the expiration date, quantity of products delivered to the retailers by indirect shipment, and sales price, respectively.


$$T_{d}^{b}\leq {exp}_{b}\;\forall b,d$$ (24)

$$0\leq \sum_{n}Q_{dr}^{bn}\leq a_{r}^{b}\;\forall b,d,r$$ (25)

$$\sum_{m}c_{md}^{b}\leq {Pr}_{dr}^{b}\leq \frac{a_{r}^{b}}{b_{b}}\;\forall b,d,r$$ (26)

Finally, constraints (28) and (29) define the decision variables of the problem.


$$Q_{sm}^{an},Q_{mr}^{bn},Q_{md}^{bn},Q_{dr}^{bn},T_{d}^{b}\geq 0$$ (27)

$$P_{m},{DC}_{d},{Tr}_{n}\in (0.1)$$ (28)

### 5. Case study

**Fig 6:**
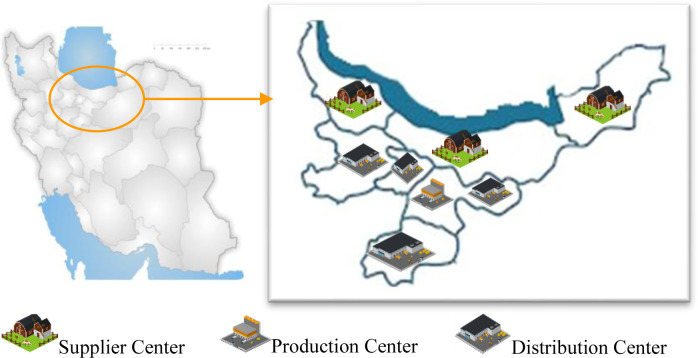
Supply chain network structure of the case study.

In this section, we implement our model on a processed food company, which we use as a case study. The goal is to evaluate the efficiency of the proposed model under real-world conditions and to address the needs of the company in question. To this end, we apply the actual data provided by the company, as well. The supply chain of this company consists of four levels: suppliers, a manufacturer, distributors, and retailers. The company manufactures three types of processed meat products: 90% beef sausage, 97% beef lunch meats, and 97% chicken lunch meats (b = 3). The products are derived from two types of raw material: chicken and beef (a = 2), each type provided by three suppliers (s = 3). Two candidate locations (m = 2) are considered in industrial towns around Tehran to establish the PCs. The finished products are distributed in Tehran, Alborz, Qazvin, and Qom provinces (d = 4). If retailers are within the coverage radius of the PC, they receive the finished products directly from the PC. Otherwise, DCs are set up to deliver products to retailers indirectly. In total, there are 12 retailers involved (r = 12). The finished products expire in 42 days (6 weeks) from the production date. Due to the perishability of raw materials and finished products, a fleet of mechanized vehicles with three different capacity levels (n = 3) are used to transport the materials and products. Finished products are stored in DCs and may even remain there beyond their expiration date. If the products are not sold during their useful life, they will decay and impose costs on the SC. The cost of product decay is the same for all DCs. Fig 6 shows a graphical image of our case study structure. Due to the importance of case study data publication, details of this study data are provided in S1 Appendix.

We use the SimaPro environmental impact calculation program to calculate all the values of Co_2_ emissions from construction and transportation by vehicles with different capacities.

## 6. Solution method

PFGP is a relatively new extension of fuzzy goal programming with different significance levels and preferences. It was conceived mainly with the goal of avoiding the restrictions of the old method to solve multi-objective problems [65]. In PFGP, the objectives are prioritized and the most essential one is assigned a higher priority grade than the others. When priorities enter the model as constraints through some equations, PFGP can be used to solve multi-objective problems with uncertain objectives and goals with different significance levels [66]. The variables and parameters of the method are as follows:

*ϑ*_*g*_ Attainment grade of objective *g*

*Up*_*g*_ Upper bound of toleration for objective *g*

*Lw*_*g*_ Lower bound of toleration for objective *g*

$P_{g}^{+}$ Positive deviation of the satisfactory level of objective *g*

$N_{g}^{-}$ Negative deviation of the satisfactory level of objective *g*

*σ*_*g*_ Expected target of objective function *g*

Thus, the general PFGP formation can be presented as follows:

$$Max\;Z=\sum_{g}\vartheta_{g}$$ (29)

s.t.

$$C_{c}(X)=(\leq \geq )0\;\forall c$$ (30)

$$\frac{1}{{Up}_{g}-\sigma_{g}}\times P_{g}^{+}+\vartheta_{g}\leq 1\;\forall g=(1,2,\ldots ,G_{i})$$ (31)

$$f_{g}-P_{g}^{+}\leq \sigma_{g}\;\forall g=(1,2,\ldots ,G_{i})$$ (32)

$$\frac{1}{\sigma_{g}-{Lw}_{g}}\times N_{g}^{-}+\vartheta_{g}\leq 1\;\forall g=(G_{i}+1,\ldots ,G)$$ (33)

$$f_{g}+N_{g}^{-}\geq \sigma_{g}\;\forall g=(G_{i}+1,\ldots ,G)$$ (34)

$$\vartheta_{g}\geq \vartheta_{g^{\prime }}\;\forall g\neq g^{\prime }$$ (35)

$$X,\vartheta_{g},P_{g}^{+},N_{g}^{-}\geq 0$$ (36)

where *C*_*c*_*(X)* is the system constraint and *f*_*g*_ is the objective function *g*. Eq (29) is the PFGP objective function and maximizes the total attainment grade of the objectives. Eq (30) presents the constraints of the original problem. Eqs (31) and (32) calculate the attainment grade for the minimization objectives, the set of which is considered to be *Ɐ g = (1*, *2*, *…*, *G*_*i*_*)*. Eqs (33) and (34) calculate the attainment grade for maximization objectives, the set of which is stated as *Ɐ g = (G*_*i+1*_, *…*, *G)*. Eq (35) specifies the objective preference and ensures that the attainment grade of the most essential objective is greater than those of the others. Lastly, Eq (36) expresses the specifications of the decision variables of both PFGP and the problem of this study. After the PFGP approach is applied, the proposed model may be defined as follows:

$$Max\;Z=\sum_{g}\vartheta_{g}$$ (37)

s.t.

(6)–(29)

$${\frac{1}{\sigma_{1}-{Lw}_{1}}\times N_{1}^{-}+\vartheta }_{1}\leq 1$$ (38)

$$f_{1}+N_{1}^{-}\geq \sigma_{1}$$ (39)

$$\frac{1}{{Up}_{2}-\sigma_{2}}\times P_{2}^{+}+\vartheta_{2}\leq 1$$ (40)

$$f_{2}-P_{2}^{+}\leq \sigma_{2}$$ (41)

$$\frac{1}{{Up}_{3}-\sigma_{3}}\times P_{3}^{+}+\vartheta_{3}\leq 1$$ (42)

$$f_{3}-P_{3}^{+}\leq \sigma_{3}$$ (43)

$$\frac{1}{\sigma_{4}-{Lw}_{4}}\times N_{4}^{-}+\vartheta_{4}\leq 1$$ (44)

$$f_{4}+N_{4}^{-}\geq \sigma_{4}$$ (45)

$$\vartheta_{1}\geq \vartheta_{2}$$ (46)

$$\vartheta_{2}\geq \vartheta_{3}$$ (47)

$$\vartheta_{3}\geq \vartheta_{4}$$ (48)

$$X,\vartheta_{g},N_{1}^{-},P_{2}^{+},P_{3}^{+},N_{4}^{-}\geq 0$$ (49)

where Eq (37) maximizes the total attainment grade, Eqs (38–45) compute the attainment grades of the objectives, Eqs (46–48) show the preference of the objectives, and Eq (49) demonstrates the characteristics of the decision variables of PFGP.

We employ the aforementioned PFGP approach due to the following points:

- Goal programming has been considered a widely-used approach to efficiently deal with models that have multiple objectives [66];
- PFGP removes the need to determine a fixed value for both the objective function and the objective function’s degree of achievement, while also taking into account any uncertainty in setting goals for the objectives [65];
- The method’s structure is simple and uncomplicated, making it easy for industry managers to comprehend [66];
- Using this approach, executives would have a simple job of modifying the priority of objective functions and observing the impact these changes make to the final values [66];
- This approach exhibits superior performance compared to the PFGP approaches developed in the past [65].

## 7. Computational results

**Fig 7:**
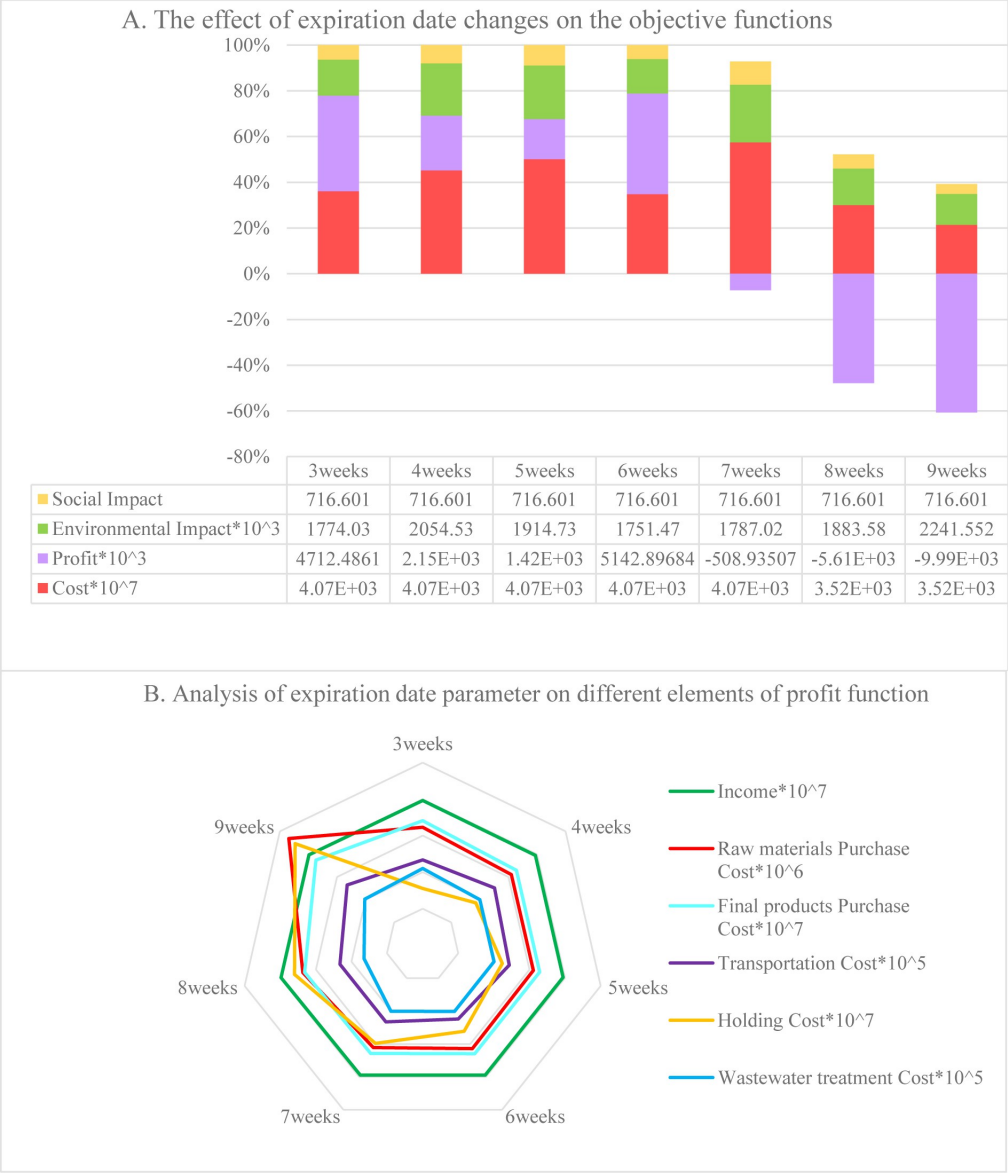
Product shelf-life effects: A) on objective functions, B) on elements of the profit function.

The proposed mathematical model was solved by the Baron solver in GAMS v25.1.3 on a computer with a 2.5 GHz Intel processor and 32 GB of RAM, and subsequently validated by multiple sensitivity analyses performed on some of the essential parameters that had a greater effect on the results. The parameters included retailer demand, constant market elasticity, price reduction coefficient in the demand function in indirect shipment, and product expiration date. As mentioned above, the expiration date of products is an essential parameter whose value is assumed to increase and decrease 1, 2 and 3 weeks to demonstrate the effects of its changes on each of the four objective functions (see Fig 7A). The effects on each element of the profit objective function are shown in Fig 7B. As expected, when the expiration date is prolonged, the duration of the order period also increases, forcing the distributors to purchase more products and causing the holding costs in DCs to increase. Hence, the products have to be delivered using higher-capacity vehicles, incurring higher transportation costs and causing greater damage to the environment in the process. When three weeks pass from the expiration date of the products, the model selects higher-capacity PCs, changing the functions of setup costs and environmental impacts. When seven weeks have passed since the expiration date, the profit drops due to high purchasing costs and longer holding times in the DCs.

**Fig 8:**
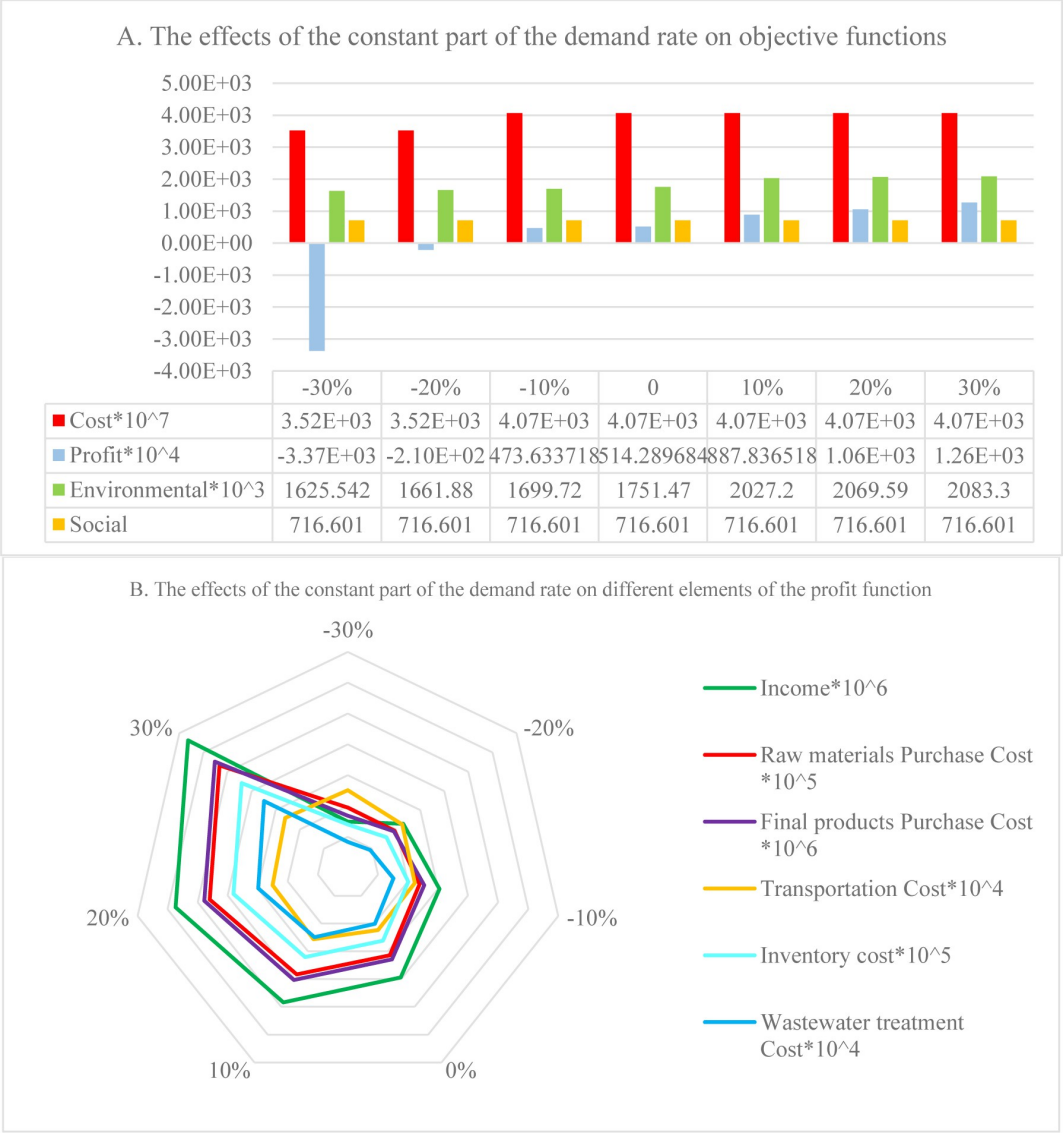
Effects of constant part of demand rate: A) on objective functions, B) on elements of the profit function.

In indirect shipment, since demand depends on the sales price, the two essential parameters are the fixed part of the demand and the price reduction coefficient in the demand function. The former was changed from -30% to +30% in 10% of the batches. The effects of these changes on the model’s objective functions and the elements of the profit function are detailed in Fig 8A and 8B, respectively. As can be seen in Fig 8A, with a 20% and 30% reduction in the fixed part of the demand, the profit function becomes a negative value due to the high costs. In both cases, the PC is different from the other cases. This also leads to changes in a) the distances of the PC from DCs and suppliers, and the distances of suppliers from each other; and b) the value of the environmental objective function. These factors also affect the costs of shipping, purchasing raw materials from the suppliers, and purchasing finished products from the PC. Furthermore, when demand increases, the quantity of DCs’ orders increase, as well. However, since the order placement time is fixed based on the expiration date, more products are stored simultaneously, causing the holding cost to increase (Fig 8B).

**Fig 9:**
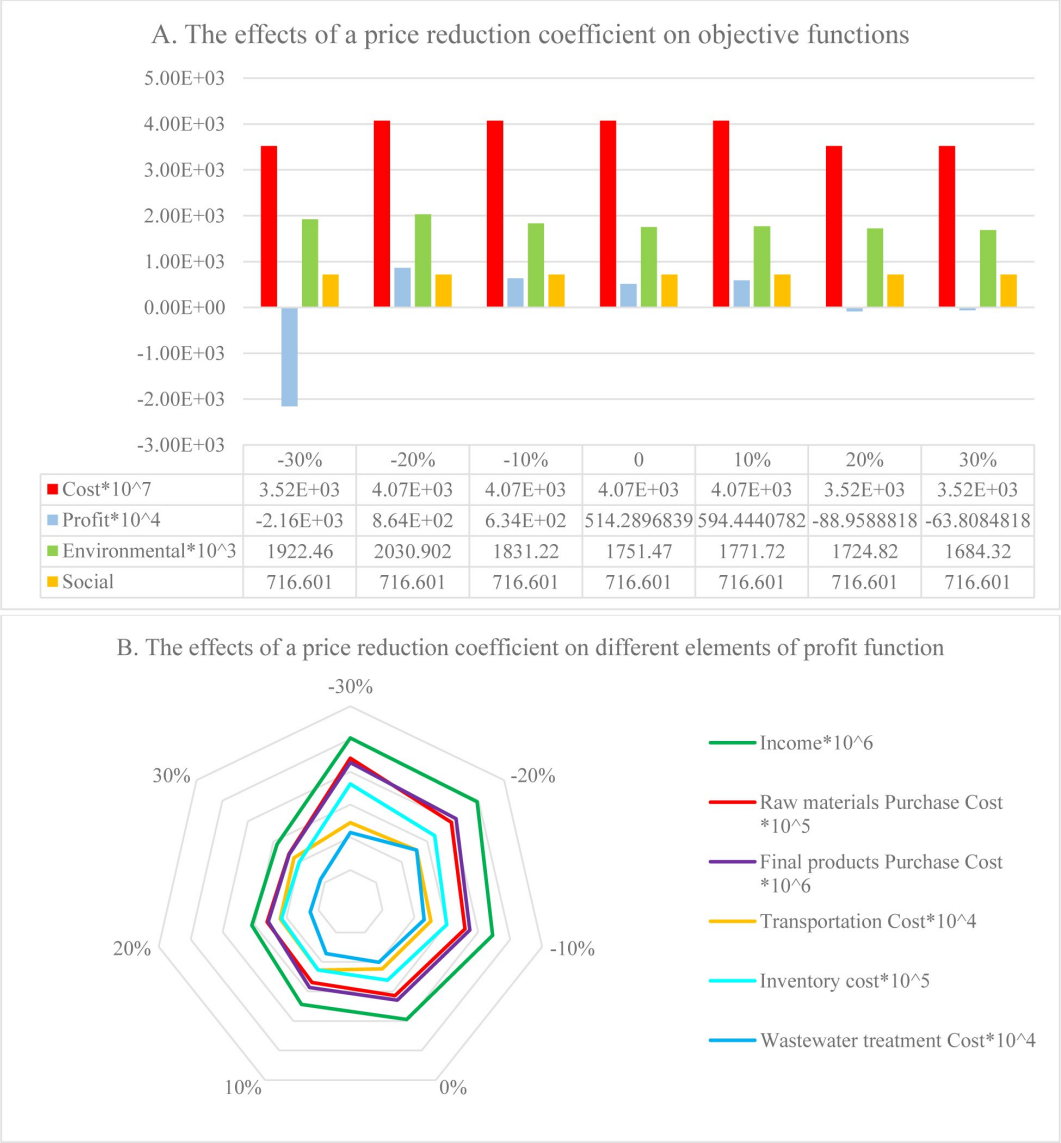
Effects of price reduction coefficient: A) on objective functions, B) on elements of the profit function.

The effects of changing the price reduction coefficient from -30% to + 30% are illustrated in Fig 9. As can be seen, when the coefficient decreases by 30% or increases by 20 or 30%, the model selects the first PC, causing a reduction in the setup costs. However, due to the increase in demand, the profit function is also decreased by 30% and turns negative. This increases the cost of purchasing the products from the PC and raw materials from the suppliers, and thereby the holding costs in the DCs. Increasing the price reduction coefficient by 20 to 30% will increase the sales price and decrease the demand and revenue. The environmental impacts also increase or decrease depending on changes in the demand and the capacity of the vehicles used to deliver the products.

**Fig 10:**
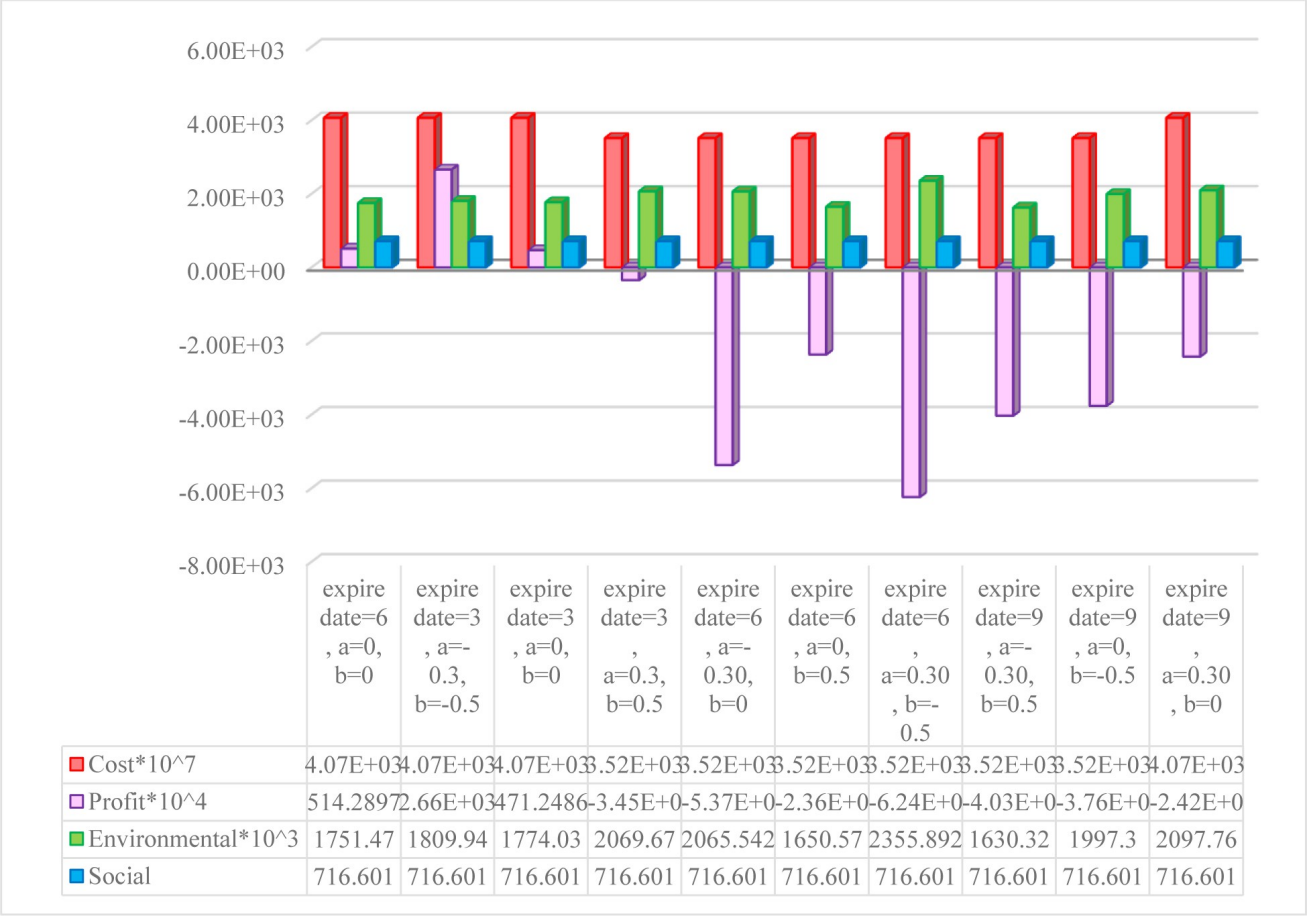
Effects of simultaneous changes in the expiration date, price reduction coefficient and constant part of the demand rate on different objective functions.

**Fig 11:**
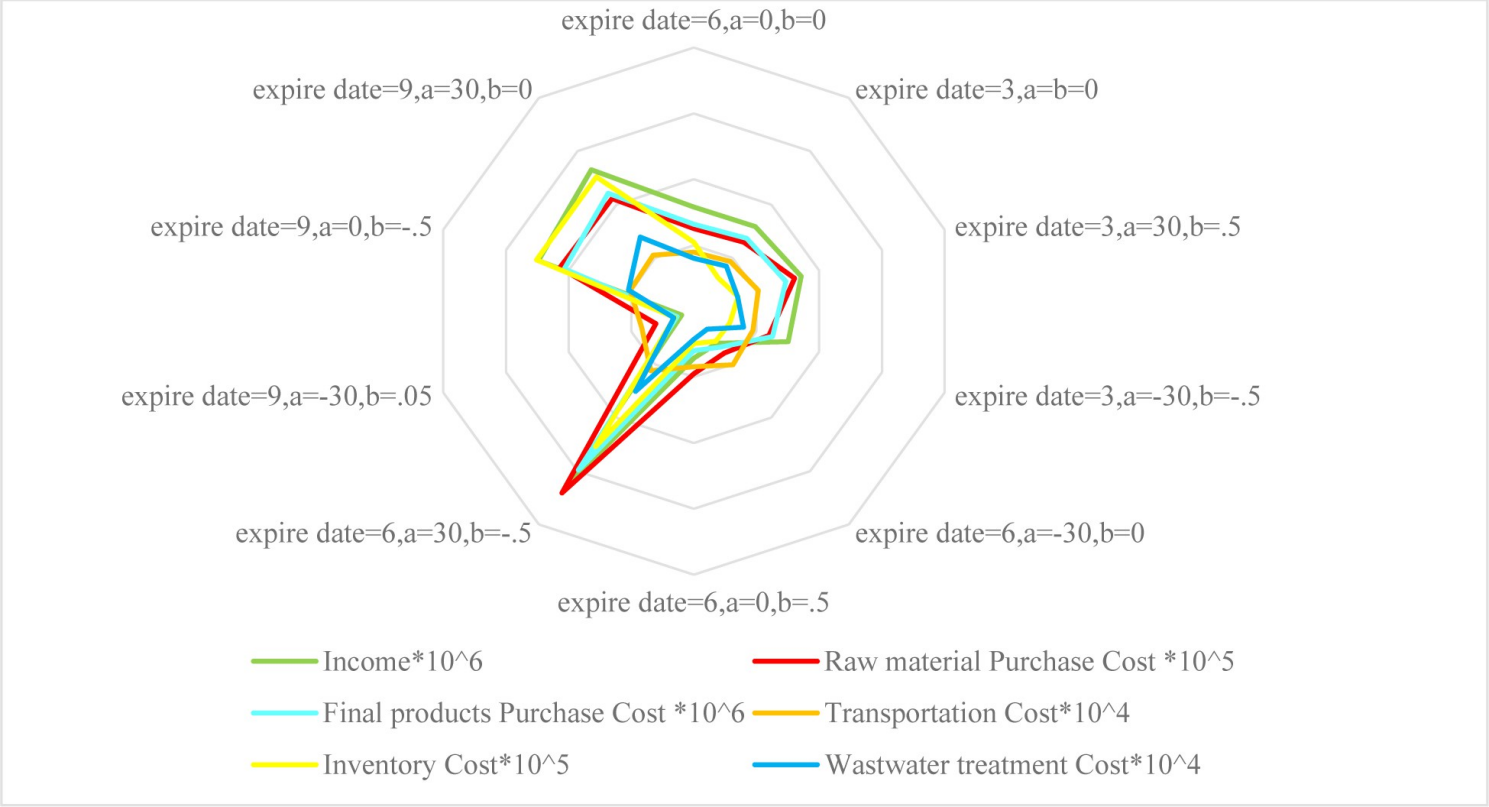
Effects of simultaneous changes in the expiration date, price reduction coefficient and constant part of the demand rate on the elements of the profit function.

Fig 10 shows the effects of making simultaneous changes to all three parameters of the objective functions. The social objective function remains unchanged because the PCs and DCs have all been established; the setup cost function differs based on each PC selection, and the environmental function differs according to the variations in the capacity of the transportation vehicles and the location of PCs. The changes of the profit function are more tangible than in the other functions as they are caused by changes in the demand, which depends on the fixed part of the demand and the price reduction coefficient of the demand function. Demand variations greatly affect the revenue from product sales and holding costs and the DCs’ purchasing costs. Therefore, these costs dramatically increase when the price reduction coefficient decreases, when the fixed part of the demand increases and causes the profit function to have a negative value, or when the expiration date is prolonged by 50%, forcing the DCs to buy and hold more products and thereby causing to a negative profit function. Fig 11 shows the effects of each of these changes on the elements of the profit function.

**Fig 12:**
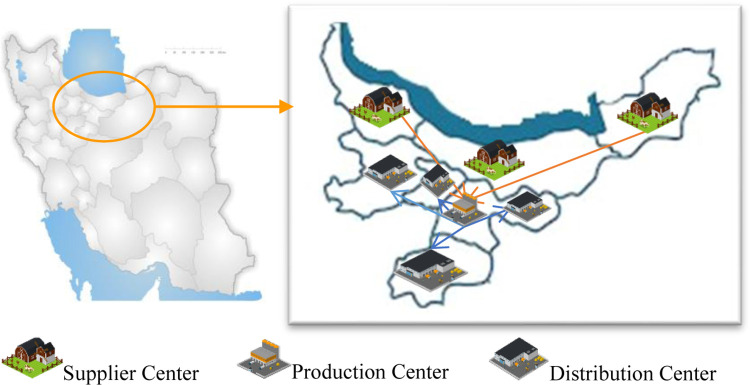
The obtained supply chain network structure of the case study.

In this case, the following locations i.e. provinces are selected to be established (Fig 12):

- Each type of raw material in the supply chain is provided by a separate supplier. The suppliers are in Golestan and Gilan.
- A PC is established in Tehran.
- Four distributors are established in Tehran, Alborz, Qazvin, and Qom.

## 8. Conclusion

In this research, we developed a multi-objective mixed-integer non-linear programming model to design a multi-level sustainable SC of processed meat products with expiration date-dependent deterioration rates and price-dependent demand. The proposed model determined important location-allocation decisions to ensure full demand satisfaction with respect to perishability, demand quantity, sales prices, and replenishment-cycle lengths. The following economic, social, and environmental objectives were defined for the proposed model: 1) minimizing the setup costs of the PC and DCs, 2) maximizing the overall SC profit, 3) maximizing the number of jobs created and regional development in areas where the PC and DCs were established, and 4) minimizing the adverse environmental impact (Co_2_ emission and wastewater generation) of the SC. The model was applied to a case study using the modeling software GAMS to illustrate the applicability of the theoretical results and was validated through several sensitivity analyses on its critical parameters including the expiration date, market elasticity, and the price reduction coefficient in the demand function. Our analyses of the data revealed that in order to fully satisfy the demand, the PC would have to be established prior to the DCs needed. The results indicated that: 1) Prolonging the expiration date increases the length of the replenishment cycle, purchasing costs and holding costs of the DCs; 2) modifying the fixed part of the demand or the price reduction coefficient in the demand function would lead to changes in the revenue and the DCs’ purchasing and holding costs; and 3) modifying some essential parameters would indeed affect the environmental impact but not the social impact. In this study, reducing the replenishment time from two weeks to one week caused a 40% reduction in product decay costs and a 25% reduction in the storage costs of distribution centers. As a result of reducing the quantity of demand per order, raw material procurement costs also decreased by about 32%. These modifications increased transportation costs by about 47%, but, overall, improved the profit objective function. The total cost of wastewater production also decreased by about 12% due to the reduced fines imposed for discharge of untreated water into the environment. The limitations of this study include real market situations which causes uncertainty of variables. The data for this research were obtained according to the standards of Iran and are limited to the present time; thus, caution should be taken when using the data or extending the result to other times and locations. Considering the limitations of and the results obtained in this study, the following directions are suggested for future research in this field.

- Given that food products can be made from both low- and high-quality raw materials, researchers may consider developing quality-dependent pricing policies for finished products.
- Since uncertainty often presents a major challenge in all areas of SCs, it is highly recommended that uncertainty be considered by researchers and incorporated into future models.
- The present study only focused on a small-scale case; however, various heuristic and metaheuristic algorithms can be developed to tackle similar problems at a larger scale.

## 9. Managerial implications

Solving a real case problem suitable with the specifications of an industry can represent both practitioners and academia valuable results. Choosing the food industry and optimizing its SC in Iran, which can gain all perspectives of sustainability, can be rewarding for managerial parties of this industry. The managerial implications concluded from the final results and sensitivity analyses are outlined as follows:

- The main costs associated with processed foods are generated by the procurement of the raw materials used for production and the purchase of finished products. Given the significant impact of this cost, even a slight reduction can have a significant effect on the final profit of the entire SC. Purchasing managers at distribution centers can estimate demand based on the demand data provided by retailers which depends on the price of finished products. Optimal ordering quantity helps reduce storage costs, while determining optimal replenishment times according to the products’ stated decay rate reduces decay costs. The manufacturer procures raw materials according to the orders it receives from distribution centers. This balance reduces the major costs of the SC and increases total profit.
- When ordering times decrease, the number of finished product shipments increase. However, with a higher number of shipments, lower-capacity vehicles are deployed. This reduces transportation costs and helps minimize the adverse environmental impact of the SC. Moreover, this strategy positively affects the situation of the wastewater generated by the production process and less untreated water is discharged into the environment.
- Since food supply chains are one of the subsets of perishable goods supply chains, the proposed model can be used for designing a SC for perishable goods with expiration dates (a.k.a. fixed shelf lives).

## Supporting information

S1 Appendix (DOCX)
