# Supplementary material for: A location-inventory model for the sustainable supply chain of perishable products based on pricing and replenishment decisions: A case study
Source: PLoS One. 2023 Jul 31;18(7):e0288915. doi: 10.1371/journal.pone.0288915 (PMC10389746; doi:10.1371/journal.pone.0288915)
Supplement: S1 Appendix — (DOCX) [file pone.0288915.s001.docx]

**Appendix A.**

In this section, we provide the case study data that we have used to solve the presented model in Section 4. The results of solving model with these parameters are presented in Section 7.

**Table A1**

Retailers' demand for different products in direct Shipment(D^b^_r_) (kg)

|  | r | | | | | | | | | | | |
| --- | --- | --- | --- | --- | --- | --- | --- | --- | --- | --- | --- | --- |
| b | 1 | 2 | 3 | 4 | 5 | 6 | 7 | 8 | 9 | 10 | 11 | 12 |
| 1 | 250 | 270 | 300 | 350 | 280 | 200 | 230 | 240 | 300 | 310 | 240 | 350 |
| 2 | 260 | 240 | 310 | 320 | 300 | 240 | 330 | 350 | 240 | 230 | 290 | 300 |
| 3 | 300 | 280 | 350 | 350 | 340 | 290 | 350 | 340 | 320 | 330 | 300 | 340 |

**Table A2**

Sales price of final products by direct shipment from Plants to Retailers(Pr^b^_mr_) (Rial×10^4^)

| 2 | | | 1 | | | m |
| --- | --- | --- | --- | --- | --- | --- |
| b | | | | | |  |
| 3 | 2 | 1 | 3 | 2 | 1 | r |
| 60 | 65 | 120 | 60 | 65 | 120 | 1 |
| 60 | 65 | 120 | 60 | 65 | 120 | 2 |
| 60 | 65 | 120 | 60 | 65 | 120 | 3 |
| 60 | 65 | 120 | 60 | 65 | 120 | 4 |
| - | - | - | - | - | - | 5-12 |

**Table A3**

Sales price of final products from Plants to Distribution Centers(C^b^_md_) (Rial×10^4^)

| 2 | | | 1 | | | m |
| --- | --- | --- | --- | --- | --- | --- |
| b | | | | | |  |
| 3 | 2 | 1 | 3 | 2 | 1 | d |
| 70 | 75 | 115 | 65 | 70 | 110 | 1 |
| 70 | 75 | 115 | 65 | 70 | 110 | 2 |
| 70 | 75 | 115 | 65 | 70 | 110 | 3 |
| 70 | 75 | 115 | 65 | 70 | 110 | 4 |

**Table A4**

Maximum Capacity of Plants (CP^b^_m_) (kg×10^3^)

|  |  | b |  |
| --- | --- | --- | --- |
| m | 1 | 2 | 3 |
| 1 | 2.5 | 3 | 2.5 |
| 2 | 2.5 | 2.5 | 3 |

**Table A5**

Maximum Capacity of Supplier (CS^a^_s_) (kg×10^3^)

|  | a | |
| --- | --- | --- |
| s | 1 | 2 |
| 1 | 3.5 | 3.5 |
| 2 | 3.5 | 3.5 |
| 3 | 3.5 | 3.5 |

**Table A6**

Maximum Capacity of Distribution Centers(CD^b^_d_) (kg×10^3^)

|  | d | | | |
| --- | --- | --- | --- | --- |
| b | 1 | 2 | 3 | 4 |
| 1 | 1.5 | 1 | 1 | 1 |
| 2 | 1.5 | 1.5 | 1 | 1 |
| 3 | 1.5 | 1 | 1.5 | 1 |

**Table A7**

Capacity of Transportation Mode (CT_n_) (kg×10^3^)

| n | 1 | 2 | 3 |
| --- | --- | --- | --- |
| CT | 2 | 4 | 6 |

**Table A8**

Purchasing cost of the row material from Suppliers (C^a^_s_)(Rial×10^3^)

|  | a | |
| --- | --- | --- |
| s | 1 | 2 |
| 1 | 65 | 250 |
| 2 | 62 | 220 |
| 3 | 63.5 | 240 |

**Table A9**

Holding cost for final products in Distribution Centers(h^b^_d_) (Rial×10^6^)

|  | b |  |  |
| --- | --- | --- | --- |
| 3 | 2 | 1 | d |
| 5 | 5 | 5 | 1 |
| 5 | 5 | 5 | 2 |
| 5 | 5 | 5 | 3 |
| 5 | 5 | 5 | 4 |

**Table A10**

Distance between Suppliers and Plants(d^a^_sm_)(km)

| 2 | | | 1 | | | a |
| --- | --- | --- | --- | --- | --- | --- |
| s | | | | | |  |
| 3 | 2 | 1 | 3 | 2 | 1 | m |
| 950 | 800 | 1000 | 170 | 150 | 130 | 1 |
| 1100 | 1000 | 950 | 180 | 170 | 120 | 2 |

**Table A11**

Distance between Plants and Distribution Centers(d_md_)(km)

| d | | | |  |
| --- | --- | --- | --- | --- |
| 4 | 3 | 2 | 1 | m |
| 190 | 200 | 170 | 110 | 1 |
| 200 | 210 | 180 | 120 | 2 |

**Table A12**

Distance between Plants and Retailers(d_mr_) (km)

|  |  | r |  |  |  |
| --- | --- | --- | --- | --- | --- |
| 5-12 | 4 | 3 | 2 | 1 | m |
| - | 220 | 250 | 210 | 190 | 1 |
| - | 260 | 270 | 200 | 210 | 2 |

**Table A13**

Distance between Distribution Centers and Retailers(d_dr_) (km)

| r | | | | | | | | | | | |  |
| --- | --- | --- | --- | --- | --- | --- | --- | --- | --- | --- | --- | --- |
| 12 | 11 | 10 | 9 | 8 | 7 | 6 | 5 | 4 | 3 | 2 | 1 | d |
| - | - | - | - | - | - | - | - | 15 | 20 | 25 | 30 | 1 |
| - | - | - | - | - | 18 | 15 | 20 | - | - | - | - | 2 |
| - | - | 15 | 30 | 20 | - | - | - | - | - | - | - | 3 |
| 20 | 20 | - | - | - | - | - | - | - | - | - | - | 4 |

**Table A14**

Number of job created and Unemployment rate in Plants (JCPP_m_) (person)

| \| m \| 1 \| 2 \| \| --- \| --- \| --- \| \| JCPP \| 25 \| 27 \| \| upp \| 13.3 \| 13.3 \| |  |
| --- | --- | --- | --- | --- | --- | --- | --- | --- | --- | --- |

**Table A15**

Number of job created and Unemployment rate in Distribution Centers (JCPD_d_) (person)

| \| d \| 1 \| 2 \| 3 \| 4 \| \| --- \| --- \| --- \| --- \| --- \| \| JCPD \| 6 \| 7 \| 6 \| 7 \| \| upd \| 13.3 \| 13.8 \| 11 \| 12.1 \| |
| --- | --- | --- | --- | --- | --- | --- | --- | --- | --- | --- | --- | --- | --- | --- | --- |

**Table A16**

Economic Value and Regional Development coefficient in Plants (EV_m_) (person)

| \| m \| 1 \| 2 \| \| --- \| --- \| --- \| \| EV \| 12 \| 12 \| \| re \| 0.6851 \| 0.6851 \| |
| --- | --- | --- | --- | --- | --- | --- | --- | --- | --- |

**Table A17**

Economic Value and Regional Development coefficient in Distribution Centers(EV_d_)(person)

| \| d \| 1 \| 2 \| 3 \| 4 \| \| --- \| --- \| --- \| --- \| --- \| \| EV \| 12 \| 11 \| 10.5 \| 8.5 \| \| re \| 0.6851 \| 0.2717 \| 0.2284 \| 0.2082 \| |
| --- | --- | --- | --- | --- | --- | --- | --- | --- | --- | --- | --- | --- | --- | --- | --- |

**Table A18**

Opening cost for Plants (FP_m_)(Rial*10^9^)

| \| m \| 1 \| 2 \| \| --- \| --- \| --- \| \| FP \| 60 \| 65 \| |
| --- | --- | --- | --- | --- | --- | --- |

**Table A19**

Opening cost for Distribution Centers (FD_d_)(Rial*10^9^)

| \| d \| 1 \| 2 \| 3 \| 4 \| \| --- \| --- \| --- \| --- \| --- \| \| FD \| 10 \| 80 \| 120 \| 9 \| |
| --- | --- | --- | --- | --- | --- | --- | --- | --- | --- | --- |
